# Gaps, Challenges, and Opportunities for Global Health Leadership Training

**DOI:** 10.5334/aogh.3219

**Published:** 2021-07-12

**Authors:** Joachim Voss, Sandul Yasobant, Anike Akridge, Edith Tarimo, Esther Seloilwe, David Hausner, Yohana Mashalla

**Affiliations:** 1 Frances Payne Bolton School of Nursing, Case Western Reserve University, Cleveland, Ohio, USA; 2 Afya Bora Consortium; 3 Center for Development Research, University of Bonn, Bonn, Germany; 4 Global Health, Institute for Hygiene and Public Health, University Hospital Bonn, Bonn, Germany; 5 Sustaining Technical and Analytical Resources (STAR) Project, Public Health Institute (PHI), Washington D.C., USA; 6 School of Nursing, Muhimbili University of Health and Allied Sciences, Dar es Salaam, Tanzania; 7 School of Nursing, University of Botswana, Gaborone, Botswana; 8 Faculty of Health Sciences, University of Botswana, Gaborone, Botswana

## Abstract

**Background:**

Global Health Leadership (GHL) programs are essential for training emerging health care professionals to be effective leaders. Synthesizing knowledge acquired through experience implementing GHL programs can inform future recommendations for GHL.

**Objective:**

To describe the lessons learned, highlighting gaps, challenges and opportunities, during implementation of two GHL capacity building programs, namely the Afya Bora Consortium Fellowship in Global Health Leadership and the Sustaining Technical and Analytic Resources (STAR) fellowship and internship program for global health professionals.

**Methods:**

A mixed methods case-comparison study was conducted, using qualitative data (expert opinion) collected from the Program Directors in order to understand the experiences of the two GHL programs. A structured response guide was used to assess the overall experience in GHL program implementation, operational challenges and reported gaps. Afya Bora and STAR have been implemented for 8 and 2.5 years respectively. Thus, the analysis reflects a snapshot of the two programs at different stages.

**Findings:**

The results reflect knowledge gained through extensive experience in implementing the two GHL programs. Afya Bora has trained 188 multi-disciplinary fellows, and 100% of the African fellows are engaged in leadership positions in government departments and non-governmental organizations (NGOs) in their countries. STAR has placed 147 participants (89 fellows and 58 interns) in more than 25 countries globally. Both programs were successful in strengthening south-south and north-south collaborations for a common goal of improving global health. Implementation of both fellowships identified room for improvement in operational procedures and financing of the programs, and highlighted knowledge and skills gaps, as well as challenges in sustainability of the training programs.

**Conclusions:**

Afya Bora and STAR have had significant impact and have contributed to changing the leadership landscape in global health. Future GHL programs should address sustainability in terms of financing, delivery modalities and domestic integration of knowledge.

## Introduction

Over the last two decades there has been greater local control and ownership of health programs, and public health efforts are increasingly being led by professionals in their own countries from medical, public health, and related fields [1]. Global health is an area for study, research, and practice that, 1) places a priority on improving quality of, and access to, health services, and on achieving equity in health for all people worldwide; 2) emphasizes transnational health issues, determinants, and solutions; 3) involves many disciplines within and beyond the health sciences and promotes interdisciplinary collaboration; and 4) is a synthesis of population-based prevention with individual-level clinical care [2,3].

With poverty, porous borders and pandemics, there is now increasing urgency to strengthen global health programs, and yet this has remained a challenge [4,5]. In many countries, health care professionals in fields such as medicine, dentistry, and nursing have become increasingly involved in global health initiatives to strengthen the response [6,7]. While pharmacists and laboratory specialists also play a critical role in the detection, prevention and control of diseases, there has been less focus on the important contributions made by pharmacists in global health, and strong leadership by laboratory personnel continues to be limited in many low- and middle-income countries [8,9]. In addition, professional fields such as communications, journalism, law, management (e.g., human resources, business, finance), and others, all have and continue to play important roles in improving global health. To sustainably collaborate in an inter-professional, multi-sectorial environment, global health professionals need to have the crucial leadership training required to build local capacity. Unfortunately, few programs have been able to incorporate leadership and situational analysis skills needed to bring evidence-based interventions and programs to scale [5,6,10,11,12].

Strong leadership requires an essential set of competencies in order to strengthen global health programs, especially during and following epidemics like Ebola and pandemics like COVID-19 [13,14]. Evidence indicates that health leadership is centred on the ability to identify priorities, provide strategic direction to multiple actors within the health system, and create commitment across the health sector to address those priorities for improved delivery of health services [13,14]. These skills are rarely included as part of research or clinical training and represent an addressable hurdle toward closing the “know-do gap” in low- and middle-income countries (LMIC) and in sub Saharan Africa (SSA) in particular.

Global Health Leadership (GHL) programs were developed in part to respond to the skills gaps identified as vital for emerging public health leaders and professionals to have sustained impact on the programs they champion. Since many of these individuals were in public health practice, not academics, programs needed to expand their content and the context of delivery beyond academic institutions in order to meet the needs of specific cohorts and local and national health systems [15,16,17,18]. In our review of available GHL programs, training content varied from simple leadership skills (such as effective communication) to more advanced training on cross-cutting leadership management skills [5,16,17,18]. While formative program evaluation data on the impact of emerging GHL programs exist, literature gaps persist in several major areas: 1) who should be trained? 2) what needs to be implemented? 2) how are programs most successfully implemented? [7,14,15].

This paper documents the lessons learned during the implementation of two GHL and capacity building programs: the Afya Bora Consortium Fellowship in Global Health Leadership (Afya Bora) and the Sustaining Technical and Analytic Resources (STAR) project [16,17,18,19]. These two programs were selected purposefully, with the aim of understanding the similarities, differences and lessons learned in implementing GHL programs under different contexts. This paper describes successes and challenges, and provides future recommendations.

## Methods

*Study type*: This is a mixed method case-comparison study, where the qualitative data (expert opinion) was collected from the Program Directors to understand their lived experiences of the Afya Bora and STAR leadership training programs.

*Selection of GHL programs*: Afya Bora has been funded by the US government through the Office of AIDS Research (OAR), Health Resources and Services Administration (HRSA), and the President’s Emergency Plan for AIDS Relief (PEPFAR) and ran from 2009 to 2020 in five African countries: Botswana, Cameroon, Kenya, Tanzania and Uganda. The program aims to provide future global health leaders with practical leadership skills that are currently not part of traditional training in the health professions. Fellows were from these countries and from the USA and China. STAR is a five-year project of the Public Health Institute, supported by the United States Agency for International Development (USAID). STAR’s goal is to strengthen the capacity of global health professionals, and organizations so that they can implement stronger programs, achieve better results, and make a bigger impact for communities and populations in need. At the time of writing, Afya Bora and STAR have been implemented for 8 years and 2.5 years respectively and this analysis reflects a snapshot of the experiences of these programs.

*Review criteria*: A structured response guide was developed to assess the responses from leaders of the programs in regard to: 1) Overall experience in the GHL program implementation, 2) Operational challenges and successes experienced during the implementation of such GHL programs, and 3) Recommendations to improve the existing programs. The reflections from the implementation of two selected cases of GHL programs are presented as findings.

## Findings

### Impetus to the Global Health Leadership Program Implementation

*Afya Bora: “We were motivated to improve the health of populations and individuals in Africa and believed that one way to do this would be by filling a gap in training. Before designing the fellowship, a meeting with more than 100 African and US health professionals was held in Nairobi to discuss what to do. Many of our African colleagues saw the need for better training of those leading public health and healthcare programs in Africa. People in health-related leadership positions had formal training in their clinical area of expertise and then were thrust into leading/managing programs without ever being taught and acquiring skills specific to effective leadership and management.”*

*STAR: “STAR was conceived by the Public Health Institute (PHI) and its partners (Johns Hopkins University, the University of California San Francisco, and the Consortium of Universities for Global Health) in response to a request from USAID. The ideas were built upon the experience and lessons learned from previous USAID-funded fellowship programs in Global Health, which had been implemented by PHI over the previous 23 years. The focus of this new fellowship program was influenced by USAID’s Journey to Self-Reliance (see: https://www.usaid.gov/selfreliance). The desire was for STAR fellows and interns to support host country governments and partners to achieve locally sustained results and to strengthen local capacities, while at the same time building their own skills to work more effectively both locally and globally.”*

### Operational issues and challenges during the implementation of such GH leadership program

#### What went well?

*Afya Bora: “The majority of the fellowship’s working group members were extremely engaged, and we had excellent representation from all countries and from both nurses and physicians. Working group members had extensive knowledge of topics and a strong commitment to capacity building. There were enough funds to bring people together and create a sense of community, trust and collaboration. This held the program together even when times were difficult from a funding standpoint.”*

*STAR: “Having highly skilled recruitment, global operations, and performance management staff has enabled STAR to operate smoothly. We are able to recruit and match Fellows and Interns to placements and onboard them quickly. We provide supportive services to the Fellows and Interns, as well as their onsite managers in host organizations throughout their fellowship and internship periods. As demonstrated during the COVID19 pandemic crisis, STAR’s global operations team has been able to pivot with rapid responses, such as repatriation when necessary, to ensure the safety of our Fellows to the greatest extent possible.”*

#### What did not go well?

*Afya Bora: “The administrative structure required for a fellowship across five African countries, the US and China was complicated, and it made decentralization of funds and administration challenging. Most of the administration was done in the US for the first several years of the program.”*

*STAR: “STAR is an ‘on-demand’ fellowship program, meaning funding is provided to the program to support fellowships one at a time, as fellowship positions are identified by the funder and the host organizations. Thus, program staff need to generate interest and demand from host organizations and from the donor in addition to recruiting, placing, on boarding, and supporting the Fellows and Interns. This led to STAR getting a slower start than would have been ideal, and it took longer than anticipated to achieve the targeted number of Fellows and Interns in the program.”*

#### How was the implementation monitored?

*Afya Bora: “Afya Bora spent considerable effort and dedicated funds to creating a robust monitoring and evaluation program with an experienced lead and a full-time staff person to collect data year-round and write reports. Retreats were also held to review the feedback and iteratively improve the modules and the overall program.”*

*STAR: “STAR staff from our performance management team, our learning team, along with our monitoring, evaluation, and learning advisor monitor and continually evaluate the fellowship and internship program at the individual and program levels. Staff check in with each Fellow on a quarterly basis to monitor progress against their learning and work objectives, and to track growth against the core competencies and milestones framework. Staff also check in with onsite managers to gain perspectives in order to intervene early in case there are any performance issues with the Fellows. The Monitoring, Evaluation and Learning (MEL) advisor conducts semi-annual and annual surveys to measure satisfaction of the Fellows, the host organization hiring managers, and the onsite managers, to ensure we are meeting the needs of everyone involved in our program. The MEL advisor is responsible for data collection, analysis, and report writing.”*

#### What were the challenges before the program implementation?

*Afya Bora: “The development of the curriculum and the development of the competencies took tremendous time and required constant attention and improvement.”*

*STAR: “Since our program is demand-driven and requires interest to be generated amongst both the host organizations and the donor in order to create fellowship opportunities, the biggest challenge initially was creating the demand that would bring funds with opportunities for fellow recruitment and placement. Additionally, developing the framework which included the GH competencies and milestones for learning and the structural foundation which included establishing HR systems (i.e. compensation packages, performance management procedures) and the global operations processes that support hiring local and third country national Fellows in LMICs took considerable time and resources.”*

#### What challenges did you experience during the program implementation?

*Afya Bora: “Funding lapses were stressful and created tensions between fellows and program leads. In some instances, fellows would apply for the fellowship through their supervisors and when the reality came for them to now enrol for the program, they would not be released. The program implementation was supposed to be rotational. However, in some instances this was not possible to rotate in the countries, which participated in the program.”*

*STAR: “The on-demand nature of STAR fellowships means we don’t have a cohort of Fellows to whom we can provide consistent training. In addition, the STAR fellowship model puts the decision-making for recruitment of the Fellows in the hands of the hosting organization and donors. This leads to a lack of internal consistency for the selection of Fellows because the final recruitment decisions are made by different people. As a result, our Fellows are very diverse in terms of their levels of experience and skill sets, their countries of origin and placements, and the subject matter focus of job descriptions. This type of program necessitates an individual learning approach. There are benefits to such an individually tailored approach for meeting the needs of the Fellows, however, there are also challenges. First, it makes it harder to foster a connection, either personal or professional, among the fellows. Second, the amount of effort it takes to determine and build out the individual learning plans, monitor the progress, follow up on problems, and foster networking opportunities is considerable and has cost implications that make demand generation for the fellowships more difficult with host organizations and donors.”*

#### Were there challenges within the organization and outside the organization?

*Afya Bora: “No major challenges. At times working group members have dropped out and we had to replace them. However, funds did not always come in a timely manner and for a fellowship that needs to recruit and pay stipends on time; this made it difficult for us. We were backed up by the University of Washington a few times with bridge funding as we awaited funds to be delivered; we would have had to stop the program without the backing.”*

#### What are you most proud of with regards to your GHL program?

*STAR: “STAR Fellows and Interns are an amazing and diverse group of global health professionals doing excellent work all over the world to improve public health systems and services to meet the needs of communities and populations.”*

#### How would you strengthen existing programs?

*Afya Bora: “Adequate funds that are granted for an extended period of time (e.g. 3–5 years) rather than year to year funding.”*

*STAR: “Establish a solid funding from an independent source. If the funding comes from the host organization there will be competing interests. The program has more control towards its objectives if they have independent funding.”*

## Discussion

COVID-19 experience highlighted global weaknesses in human resources for health, financing, effective communication and leadership. This has raised awareness that there is need to strengthen training of health and allied professionals on leadership for global health programs. By exploring the motivation for creation, key elements in program design, and implementation challenges and successes of two leading GHL programs, we identify insights that can serve to inform ways to bridge the current gaps in leadership development for global health professionals.

### Successes

Both programs have indicated successes in several areas including program development, identification of partner/host countries and institutions, employing competent and experienced faculty and being able to smoothly recruit and deploy fellows to the host organizations or training sites. Over the past eight years, Afya Bora has trained 188 fellows and the survey in 2018 indicated that 100% of the African fellows have returned to their countries where they are engaged in leadership positions in government departments and non-governmental organizations (NGOs) (Afya Bora program Overview 2019). STAR, as a newer Global Health fellowship program operating for 2.5 years, has placed 147 participants (89 fellows and 58 interns) in more than 25 countries globally as well as in Washington, DC—many of whom are host country nationals. STAR host sites include Ministries of Health, non-governmental organizations, UN Agencies, USAID Missions and USAID Washington Headquarters. As both programs continue to have an impact on various disciplines and multiple sectors within an interdependent health system, they underscore the importance and opportunity of making GHL programs multi-disciplinary and inter-professional in nature [14,15].

#### (1) Foundation of Core GH Competencies

Foundations of core GH competencies are presented in ***Table 1***. From their inception both Afya Bora and STAR prioritized developing a core set of distinct and multi-disciplinary competencies aimed to bolster global health leadership and practice. This development process involved literature reviews, validation, creating relevant content and establishing processes for measuring participant growth. In the case of Afya Bora the core competencies included 12 skill-based core competencies and 5–7 skills were related to each competency in the “skills logbook” that the fellows completed by the end of the program. STAR’s GH competency framework includes eight core domains that focus on leadership development, including a combination of power skills and essential perspectives. In addition to the core competency domains the team identify 8–10 knowledge-based content areas (i.e., HIV, TB, MCH) and 8–10 skills-based technical areas (i.e., epidemiology, supply-chain management) where fellows could also focus their training based on their job requirements. The core domains were taught using mentorship groups, virtual exchanges and online learning modules [19]. Opposed to a skill log, STAR developed a milestones model based on the Accreditation Council for Graduate Medical Education (ACGME) milestones framework to capture the “level” of competency and opportunities for growth among STAR participants [20]. Throughout both programs, participant data has been analysed to understand where the key learning gaps of global health professionals are as they are entering into the program.

**Table 1:** Summarized findings of the case-comparison of GHL programs.


FEATURES	AFYA BORA GHL PROGRAM	STAR GH FELLOWSHIP PROGRAM

*Evolution of the Program*	Brain drain occurred when African professionals received formal advanced degrees in US, Europe and Australia and did not return back to their home countries. Leadership taught in the formal medical and nursing curricula were not adequate. Many health and HIV organizations in Africa relied on Expatriates to fill the positions because of a lack of training to manage such programs within the country. In 2009, 100 African and US leaders met in Nairobi, Kenya to define the original intent of a Global Health Leadership Training. Major issue identified was that while many clinicians were highly skilled professionals, most lacked leadership skills and experiences to manage regional or national health programs. Formation of working group to develop curriculum with equal representation of African and US members. Identification of attachment sites with capacity to mentor fellows in Botswana, Kenya Uganda and Tanzania was carried out by the working group. Development of a selection (matching) process of fellows from initially Uganda, Kenya, Tanzania and Botswana through the local national universities. A six months project pilot in 2010 was conducted to understand and improve the implementation processes, test the learning content, and shape the outcomes expectations.	Evolved from a 23-year legacy of global health fellowship programs funded by USAID, which aimed to provide opportunities for Americans and US permanent residents to work in technical positions within USAID. Established as five-year cooperative agreements that begun with the Population Leadership Program (PLP), which then evolved to Global Health Fellows Program I and then Global Health Fellows II. Each subsequent program continued to refine approaches and track records to recruiting and supporting diverse fellows and interns, while expanding its strategies to include a wider range of highly qualified talent across the global health pipeline as well as and placement sites. In 2018, under the USAID Forward initiative during a period that focused heavily on the journey to self-reliance, USAID’s vision, reflected in the STAR Program emphasized two major themes: (1) preparing diverse US (United States) and developing country GH professionals to innovate lasting solutions, based on deep mastery of program implementation, research and analytic skills that are honed during field experience and; (2) fostering effective, empowered, sustainable collaborations among US and LMIC academia, and other relevant groups. STAR aims to strengthen the capacity of global health professionals, organizations, and companies to implement stronger programs, achieve better results, and make a bigger impact in the global health field. Position based opportunities are available on a rolling basis to qualified candidates globally. Due to the diversity of participants, their technical skills and geographic placements and the unique reality of rolling admissions, a cohort model was not feasible thus, the fellowship developed tailored individual learning opportunities for both the participants and the host organizations. Developed STAR competencies and milestones framework and associated tools to standardize the core skills required of GH professionals and help individuals identify their need, leverage resources to address learning gaps, and track milestones using a common approach.

*Target Population*	Nurses, physicians, public health professionals, pharmacists, ethicists, and lawyers.	STAR targets diverse early career to senior global public health professionals from the US and from low- and middle-income countries.

*Salient Activities*	Attend three in-person didactic training meetings and complete online modules. Be attached at a national training site to be engaged in leadership activities through team mentorship. Participate in meetings with primary and site mentors and joint meetings. Complete M&E activities and logbook on a regular basis. Planned orientation, mid fellowship and final meeting held to enable familiarisation of participants to the program, sharing and presentations of projects conducted by fellows for constructive feedback. Complete required report at the end of the program and present at each meeting. Communicate regularly with site and national mentor.	Work-based fellowship at USAID, Ministry of Health or NGO Performance Management support Protected time for individualized, deliberate/targeted learning opportunities (10% -4 hours/week) Individualized Learning plan with learning objectives and relevant learning activities Mentorship and coaching (both one-on-one and group) Peer-to-peer learning and network building

*Competencies/Core Content*	Twelve training modules (leadership, communication, monitoring and evaluation, responsible conduct of research, research methods, project management, implementation science, HR/budgeting, grant/manuscript writing, health informatics, policy governance and 57 competencies are listed related to 11 modules. At least 70% achievement is expected and the Logbook has to be submitted at the Mid- and final meeting to the M&E coordinator. Experiential leadership learning occurred during the attachment site periods where fellows learned from real-world leadership experiences.	Eight core skills-based competency domains (development practice, communication and interpersonal effectiveness, cross-cultural practice, capacity strengthening, global burden of disease, ethics, health equity and social justice, and gender equity). There also are twenty+ elective technical and content-based competencies that are relevant depending on the job description and learning objectives of the participant. For each competency domain, milestones defined across five levels (inquiring, understanding, practicing, leading and advancing) and a set of measurable targets for continued advancement of knowledge, attitudes, and skills, which will enable tracking and assessment of learning for all participants.

*Key Operational Issues*	Finding accommodations for the fellows during the meetings. Transportation for the fellows and assuring that every fellow had a workable computer. Using university facilities to house the meetings. Online learning with slow broadband connections. Assuring safety of fellows during public unrest at the sites and during the meetings. To organize travel for such a diverse group of people three times a year is a major challenge.	Balancing meeting the needs of the fellow, the host organization and funder (in most cases, host and funder is the same). Managing a global staffing sub-contractor to hire and be legally responsible for internationally placed local and third country national fellows. Individualized learning model is both time and labour intensive and participants have varying needs from academic to applied technical. Delays to on boarding of fellows due to security clearance and working visas.

*Key Challenges*	Initial training was conceptualized as a two-year training but was not realistic and scaled back to a one-year program. Decentralized fund distribution was challenging and caused delays in payments to the fellows. Financial management mostly through University of Washington for a majority of the program. Ensuring that the modules had an African and an American instructor to teach the content. Validating the content with the fellows in terms of relevance and usability. Funders were slow to send the funding which made it necessary the University of Washington helped bridge the funding at times. The governments of Botswana, Uganda, Kenya and Tanzania did not begin as initially conceptualized to fund portions of the program and expand the scope of the project. Focus on HIV-related issues limited the scope of the training program initially. Some fellows could not be released 100% so requirements of the fellowship had to be modified. E.g. From fulltime to part time.	Navigating a balancing act-managing the function of a staffing mechanism with the ethos of a fellowship Adapting to the complex reality of being a US-based organization with fellows placed and employed locally in multiple countries but without local established registration. This challenge required contracting a global staffing sub-contractor. STAR recruitment team does not make final fellow selection, decision is done jointly with host organization hiring team thus recruitment team composition and selection determinations are different for each fellow. This makes it difficult to plan learning until they are on-board. One of the reasons STAR designed individualised learning model. Cannot physically bring fellows together due to geographic vastness of their placements, thus restricted to virtual learning programming Measuring achievement of the competency milestones-currently have guided questionnaire that guides staff in placing fellows for the baseline and the mid-line is self-report.

*Key Successes*	Successful training of 179 fellows and placements in Cameroon, Kenia, Botswana, Tanzania, China, and Uganda. High level of satisfaction with the program and high level of career advancements due to the program. Major impact in implementation of national health programs, education, and leadership.	Successful placements of over 79 Fellows and approximately 55 Interns in 20+ countries and Washington, DC. High level of satisfaction among Fellows and Interns about their placements and learning opportunities. High level of satisfaction among hiring managers and onsite managers at hosting organizations with the Fellows and Interns they received.

*Key Recommendations*	The curriculum content is highly relevant and has been found effective and applicable. Fostering South-South collaborations has established a network of highly collaborative interprofessional global health leaders that have impacted the planning, delivery, and implementation of much improved healthcare. Research is needed to determine the gaps in training after they have been multiple years in their leadership roles. The demand far outstripped the supply of fellowship positions and more such leadership models are needed to help the human infrastructure of global health leaders. Political support from within the countries is needed to recognize the benefits of better trained global health leaders and eliminate the gender inequity in many African countries. Nurses need an expanded scope of advanced practice to fully assume leadership positions in most African countries.	For nascent fellowship programs, be clear about your objectives; identify the purpose, the gap it is filling and then stick to it as closely as possible within the parameters of the of funders. Establish a good and solid source of funding from an independent source that fully covers project start up, as well as operating costs of the fellowship program. This will ensure more control towards achieving objectives. Balancing the needs of the fellow and the host organization (and the funder)- Our process is designed to meet the needs of the funder (host organization) first, which sometimes can dilute the ability to deliver upon objectives of the fellowship. Employ cohort model where possible- Easier to administer, promotes peer to peer collaboration for fellows and allows for consistent delivery of high-quality core learning content.


#### (2) Strengthening local leadership and global bridge builders

Afya Bora’s success in inter-professional training were the program’s outputs of nurses, physicians, and public health professionals who attained equal standards of exposure and experience which will in future enhance team building and cohesiveness among health professionals. For STAR, many of the host country nationals often serve as internal consultants and as cultural brokers between the Ministry of Health and the US government. One of the great successes is being able to nurture local leadership that is both conversant with local context, can navigate the complexities of the donor environment and can communicate effectively to achieve desired results. Both programs have indicated successes in strengthening north-south and south-south collaboration, which enhanced the establishment of strong and functional national, regional and global networks that will facilitate sharing of experiences, ideas and resources for a common goal of improving global health.

#### (3) Providing opportunities to develop applied leadership skills

Health systems are complex and continuously changing over time; they are situated in a variety of contexts and cover a range of service levels [21]. GHL training should provide opportunities to learn strategic leadership skills that fit different health systems and should enhance technical, cognitive and emotional competencies of the future leaders. A 360-degree leadership approach might help future leaders to lead from any position and at any level of healthcare system (such as policymaking or clinical service) while relying on the core leadership characteristics [22,23,24,25]. This approach could be incorporated into GHL trainings to teach future leaders to face different health system challenges through introspection and mindfulness.

#### (4) Virtual Learning

While the majority of STAR Learning was delivered through virtual programming due to the considerable distances between participants, Afya Bora, a regionally based program, used a hybrid approach of in-person and online learning. However, with the unanticipated COVID-19 pandemic, all learning has been required to shift to virtual, thus creating an opportunity to develop dynamic learning content that addresses the gaps, reduces costs and enables participants to connect globally.

### Gaps in GHL training programs

#### (1) Knowledge and Skills

While both fellowships developed robust programs that aimed to prepare their multi-disciplinary participants for a world in applied global health leadership, some skills gaps were observed as participants entered into the program. Among the key findings from implementing the STAR baseline competency assessment were knowledge/skills gaps related to gender equity, ethics and health equity and social justice. In addition, participants identified several technical and content areas in which they desired training to be more effective in their roles. Similarly, as Afya Bora evolved, the needs for consideration on some critically relevant courses were observed. ***Table 2*** below includes both the core competencies and many of the additional gaps identified or observed by both programs. Future training for the GHL should aim to standardize content in the modules and emphasis should be given to equal prioritization of the technical content and leadership development courses [21,26,27].

**Table 2:** Competencies and Gaps of STAR and Afya Bora.


STAR COMPETENCIES	TRAINING GAPS	AFYA BORA COMPETENCIES	TRAINING GAPS

Development Practice Cross-Cultural Practice Global Burden of Disease Capacity Strengthening *Gender Equity *Global Health and Social Justice *Global Health Ethics Inter-personal Communication	Effective communication Negotiation skills Diplomacy skills Public speaking Data Analysis Tableau, R, STATA and Python Data Visualization (intermediate to advanced) Operational research (beginner to advance) Academic/Scientific and program writing skills to support dissemination **and** publishing of results Health Policy Health Policy development Skills to develop effective SOPs Translating technical concepts for policymakers Health Financing Health Financing reform Mentorship skills (for individuals and teams)	These were the main competency areas with each 5–7 sub competencies: Leadership Communication Monitoring and Evaluation Research Conduct Research Proposal Writing Human Resources and Budgeting Grant Writing Institutional Research Project Management Global Policy and Governance Health Informatics Project Management	More time to practice in-class writing Qualitative data analysis methods Cost effectiveness analysis Methods of implementation science research Team building exercises Strategic planning Grant development and review process Conflict resolution skills Communication through the media Communication etiquette Grievances management and handling disciplinary workplace issues


* Competencies in which majority of participants came in with limited knowledge and exposure.

#### (2) Sector Gaps

While there is a tendency to believe that global health is the responsibility of health professionals, global health issues, such as emerging and re-emerging diseases, affect all economic sectors and life in general. Both programs boasted a high proportion of clinical professionals (i.e., physicians and nurses) or public health professionals (i.e., technical advisors, implementation scientists). This sectorial gap is also reflected in the literature on global health, where a focus among professionals like lawyers, law enforcing institutions, agriculture, wildlife is not seen. In order to align global health workforce development with the UN Strategic Development Goals, future global health programs should be developed to include exposure to these sectors [28].

### Challenges

#### (1) Operational

Both programs have indicated issues of concern in the implementation of the programs and include those related to operational procedures (program development and identification of relevant content, recruitment of experienced and competent faculty, determination of effective delivery modes, recruitment of fellows, monitoring and evaluation) and financing. The program-related operational issues, such as delay in fellow response, technical issues such as broadband connection, funds mobilization have been reported and pose major challenges in effective and timely completion of assignments and Monitoring & Evaluation (M&E) reports [29].

#### (2) Funding

Donor fund disruptions and the lack of unrestricted funds created stress and uncertainty on GHL program implementation. While it is recognized that there is a funding transition towards domestic financing for global health, sustaining these efforts will depend on creating the systems that ensure a core set of funds are available to support basic administration and the fellows’ livelihood throughout the training [21,30]. Future GHL programs are encouraged to develop realistic sustainable funding models at the outset that will ensure streamlined, regular and predictable financing, perhaps adopting a hybrid funding structure that includes core funds to support the underlying infrastructure and supplementary funds focused on the fellows. Both programs also required significant technical and programmatic support during the start-up period, funding to “build” a rigorous program must also be considered by donors. Lastly, as seen with the M&E paper, that lack of funding for “impact” evaluation post the award period means that there is limited ability to codify and understand the true impact of these programs on the ultimate goal – global health leadership.

#### (3) Sustainability

Sustainability of programs depends on three key areas including academic, technical and financial sustainability. This case report has indicated clear academic sustainability of health professionals trained in global health leadership. The content of the programs have built capacity of the alumni in leadership skills including communication, implementation science, grant writing, research and publication skills, project management, human resources and budgeting. These skills are fundamental in developing a pool of competent human resources for addressing global health challenges. In addition, faculty from the U.S. teamed with faculty from the African institutions in training the fellows. This form of collaborative teaching built capacity among the faculty across all nine institutions in Africa and U.S. for training global health professionals from different countries and different disciplines. Capacity has also been built in writing fundable research projects and providing an opportunity to network across national, regional and international programs. With these successes in mind, future GHL should target understanding the dynamics of global to local governance and sustainable funding to improve GHL training programs that will benefit future leaders.

**Figure 1:**
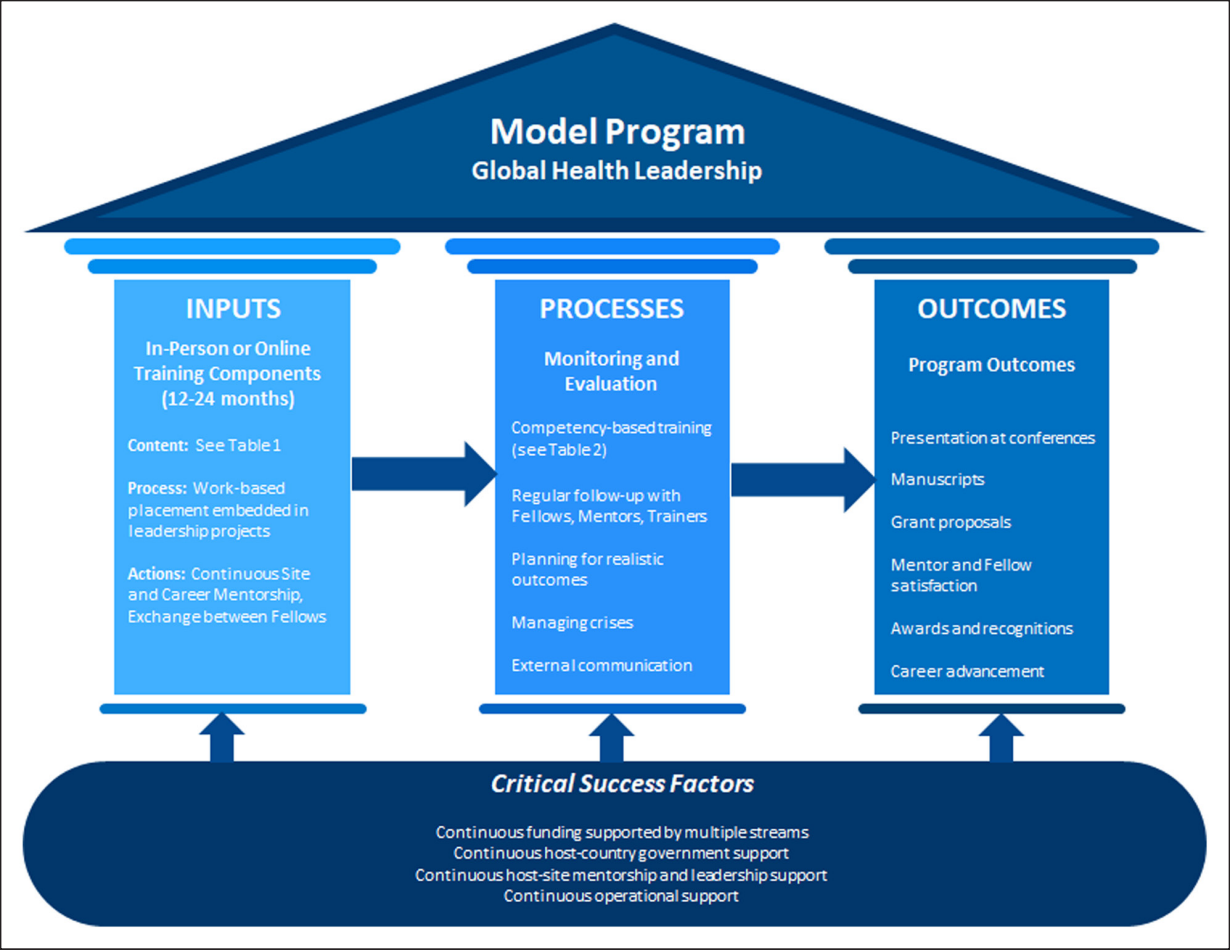
Model Program Components for Global Health Leadership Program. The proposed Model Program of Global Health Leadership informed by the experiences of STAR, Afya Bora and reinforced by the literature, presented above and the three key pillars for a successful Global Health Leadership program are borrowed from the Donabedian’s Process Improvement Framework which measures overall quality and align improvement work in health delivery settings [31]. This framework is based on input, process and outcome components. Details about the (input) educational content can be found in Table 1 of this manuscript, while content of the (process) competency components can be found in Table 2. The success metrics of the model stand on the continuous financial, management, mentorship and political support of the implementing partners. However, flexibility is allowed depending on the programmatic design in each of the model components and depends on the available resources, goals of the program, and intentions of the program partners. The length of a program depends entirely, if the fellows are familiar with the host institution or not. If they are not familiar, a longer fellowship increases the value and outcomes of the fellow to the organization.

*Based on the successes and gaps of two global leadership programs (STAR and Afya Bora) we have developed a model graph to include our key recommendations for future global leadership programs (see **Figure 1**)*.
